# *SELP* Asp603Asn and severe thrombosis in COVID-19 males

**DOI:** 10.1186/s13045-021-01136-9

**Published:** 2021-08-16

**Authors:** Chiara Fallerini, Sergio Daga, Elisa Benetti, Nicola Picchiotti, Kristina Zguro, Francesca Catapano, Virginia Baroni, Simone Lanini, Alessandro Bucalossi, Giuseppe Marotta, Francesca Colombo, Margherita Baldassarri, Francesca Fava, Giada Beligni, Laura Di Sarno, Diana Alaverdian, Maria Palmieri, Susanna Croci, Andrea M. Isidori, Simone Furini, Elisa Frullanti, GEN-COVID Multicenter Study, Alessandra Renieri, Francesca Mari

**Affiliations:** 1 Medical Genetics Unit, University of Siena, Policlinico Le Scotte, Viale Bracci, 2, 53100 Siena, Italy; 2 Department of Medical Biotechnologies, Med Biotech Hub and Competence Center, University of Siena, Siena, Italy; 3 Department of Mathematics, University of Pavia, Pavia, Italy; 4 DIISM-SAILAB, University of Siena, Siena, Italy; 5 National Institute for the Infectious Diseases “L. Spallanzani”, Rome, Italy; 6 Stem Cell Transplant and Cellular Therapy Unit, University Hospital, Siena, Italy; 7 Istituto di Tecnologie Biomediche – Consiglio Nazionale delle Ricerche, Segrate, MI Italy; 8 Genetica Medica, Azienda Ospedaliero-Universitaria Senese, Siena, Italy; 9 Department of Experimental Medicine, Sapienza University of Rome, Rome, Italy

**Keywords:** COVID-19, Thromboembolism, Thrombus, Venous thromboembolism, P-selectin, Anti-selectin P monoclonal antibodies

## Abstract

Thromboembolism is a frequent cause of severity and mortality in COVID-19. However, the etiology of this phenomenon is not well understood. A cohort of 1186 subjects, from the GEN-COVID consortium, infected by SARS-CoV-2 with different severity was stratified by sex and adjusted by age. Then, common coding variants from whole exome sequencing were mined by LASSO logistic regression. The homozygosity of the cell adhesion molecule P-selectin gene (*SELP)* rs6127 (c.1807G > A; p.Asp603Asn) which has been already associated with thrombotic risk is found to be associated with severity in the male subcohort of 513 subjects (odds ratio = 2.27, 95% Confidence Interval 1.54–3.36). As the *SELP* gene is downregulated by testosterone, the odd ratio is increased in males older than 50 (OR 2.42, 95% CI 1.53–3.82). Asn/Asn homozygotes have increased D-dimers values especially when associated with poly Q ≥ 23 in the androgen receptor (OR 3.26, 95% CI 1.41–7.52). These results provide a rationale for the repurposing of antibodies against P-selectin as adjuvant therapy in rs6127 male homozygotes especially if older than 50 or with an impaired androgen receptor.

**Supplementary Information:**

The online version contains supplementary material available at 10.1186/s13045-021-01136-9.

## To the Editor

It is now widely recognized that COVID-19 is a systemic disease, characterized by dysregulation of the immune system and by a hypercoagulable state [1]. The bases of this prothrombotic susceptibility remain until now elusive, even if it is evident that host genetic factors largely contribute to COVID-19 phenotypic variability. Rare variants of genes involved in adaptive immunity have been identified in Mendelian forms of COVID-19, where the presence of one rare mutation leads to a severe COVID-19 phenotype segregating in the family following a classic Mendelian inheritance pattern [2]. Among common genetic factors, the protective role of the 0 blood group has been identified, at least in part possibly due to von Willebrand factor (vWF) destabilization protecting from thrombosis [3]. We have also shown that longer polyQ repeats (≥ 23) in the androgen receptor (AR) predispose to severe COVID-19 outcome due to reduced testosterone anti-inflammatory and anti-thrombotic effect [4].

The P-selectin (*SELP*) gene encodes a cell adhesion molecule mediating the interaction of activated platelets on endothelium with leukocytes and playing a key role in thrombosis [5, 6]. Furthermore, significantly increased P-selectin and other prothrombotic biomarkers concentration in plasma samples of severe COVID-19 patients compared to healthy controls has been recently reported [7, 8].

Among *SELP* variants, the Asp603Asn functional polymorphism (rs6127; c.1807G > A-previously reported as Asp562Asn or Asp541Asn) has been associated with thrombotic risk in various conditions [9, 10]. The polymorphism, together with other coding polymorphisms, has indeed been shown to affect the binding of P-selectin to its ligand on leukocytes, possibly making the protein more efficient at recruiting leukocytes to the endothelium [10].

**Fig. 1:**
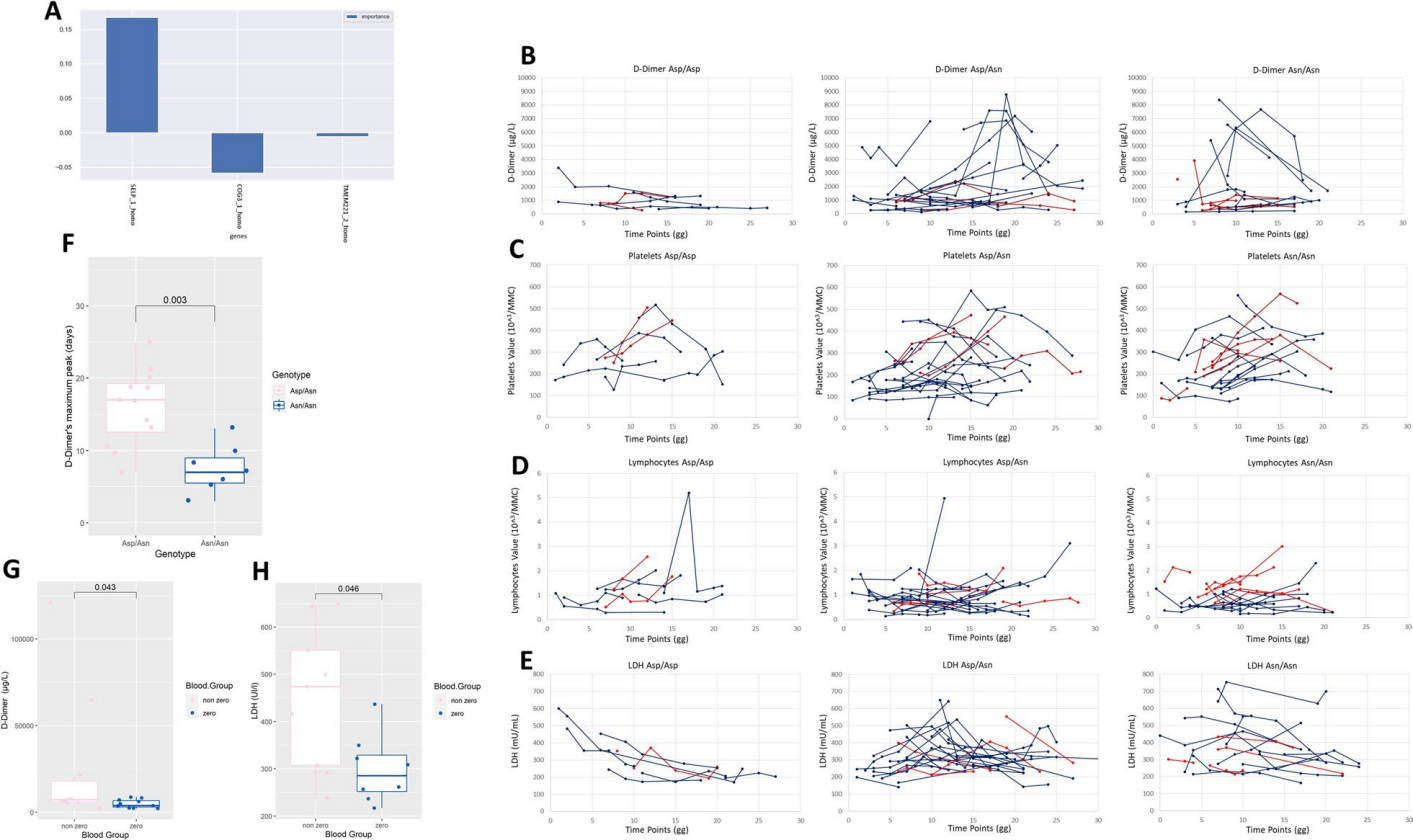
Homozygous genotype Asn/Asn at the polymorphic locus Asp603Asn (rs6127) is related to severity and to D-dimer pick. **a** Selection of *SELP* gene as relevant for severity. LASSO logistic regression on Boolean representation of homozygous common bi-allelic polymorphism of autosomal genes in males is presented (see paper Picchiotti et al. 2021 for complete representations)^20^. The LASSO logistic regression model provides an embedded feature selection method within the binary classification tasks (severe vs mild). The upward histogram means positive weights, i.e., the specific variant at the specific locus (feature) contributes to severity of COVID-19. *SELP*_1_homo = homozygous genotype Asn/Asn at the polymorphic locus Asp603Asn (rs6127). The downward histograms mean negative weights, contributing to mildness of COVID-19. *COG3*_1_homo = homozygous genotype Ser/Ser at the polymorphic locus Leu825Ser (rs3014902). *COG3* gene encodes for a vesicle docking protein involved in viral trafficking. *TMEM221*_2_homo = homozygous genotype Ala/Ala at the polymorphic locus Thr66Ala (rs4808641). *TMEM221* gene encodes for a transmembrane protein. **b**–**e** Longitudinal laboratory data related to thrombosis and severity. Linear graphs of four laboratory values: D-dimer μg/L (**b**), platelets 10^3^/mmc (**c**), lymphocytes 10^3^/mmc (**d**), LDH UI/L (n.v. 135- 225 UI/L) (**e**). As expected, the Asn/Asn homozygous genotype was over-represented (36.53%). Values are reported on the Y-axis. In each graph, the time point “0” (X-axis) represents the day of onset of COVID-19 symptoms. Each line represents each severe hospitalized patient (see methods). Each point represents the different time point (day) in which the different values have been measured. Patients aged ≥ 55 years are indicated in blue, while patients aged < 55 years are in red. From left to right patients having Asp/Asp homozygous; Asp/Asn heterozygous; and Asn/Asn homozygous genotype. Older patients only (blue) and Asp/Asn-Asn/Asn genotype only show the D-dimer pick. Accordingly, older patients of these two genotypes have more platelet consumption and higher LDH values. A total of 51 patients have been included in **c**. Among these, 23 patients have a platelet count value below 150 × 10^3^/mmc: 9 with the Asn/Asn genotype, 13 with Asn/Asp and 1 with Asp/Asp. A total of 48 patients have been included in panel D. Among these, 27 patients have lymphocyte count below 0.9 10^3/mmc: 4 Asn/Asn, 19 Asn/Asp and 4 Asp/Asp. A total of 50 patients have been included in panel E. Among these, 44 have LDH values above 225 UI/L: 16 Asn/Asn, 23 Asn/Asp and 5 Asp/Asp. **f** The D-dimer pick is earlier in the Asn/Asn (median = 7.5 days) than the Asp/Asn genotype (*p* = 3 × 10^–2^ by Mann–Whitney test). Box plots of patients with D-dimer values above 2000 µg/l were represented. Only Asp/Asn (light blue) and Asn/Asn (pink) genotypes are represented because patients with the Asp/Asp genotype do not have the pick and do not show values above 2.000. A total of 47 patients have been included in panel B. Among these, 20 patients show D-Dimer values above 2000 µg/L: 7 Asn/Asn, 12 Asn/Asp and 1 Asp/Asp. **g**, **h** The nonzero group associates with higher D-dimer (**g**) and LDH values (**h**). Severe hospitalized patients with 0 blood group = light blue; non-0 blood group = pink in box plots

Within the Italian GEN-COVID cohort, we applied an ordered logistic regression to the clinical WHO gradings, stratified by sex and adjusted by age in order to define severe and mild patients (see Additional file 1: Supplementary file). We then tested by LASSO logistic regression different combinations of coding polymorphisms in homozygous state and found that the *SELP* rs6127 polymorphism correlates with severity only in the subcohort of males (Fig. 1a; Table 1a; Supplementary file; data on females not shown). The genotypic frequencies of the polymorphism in severe and mild patients were confirmed to be in Hardy–Weinberg equilibrium; the minor allele frequency in our cohort was similar to that reported in the European (non-Finnish) population in the gnomAD database (56.2% vs 55.8%) (https://gnomad.broadinstitute.org/).

**Table 1:** Chi-square test in male cohort calculated for all ages (a); for age ≥ 50 years (b); and combination of *AR* poly-Q ≥ 23 and D-dimer value (c)

a	Severe (%)	Mild (%)	Marginal row totals
*Chi-square test in male cohort (all ages)*			
Asn/Asn genotype	90 (38.14)	59 (21.30)	149
Asp/Asp and Asp/Asn genotype	146 (61.86)	218 (78.70)	364
Marginal column totals	236 (100)	277 (100)	513 (grand total)

b	Severe (%)	Mild (%)	Marginal row totals
*Chi-square test in males* ≥ *50 years*			
Asn/Asn genotype	73 (39.25)	40(21.05)	113
Asp/Asp and Asp/Asn genotype	113 (60.75)	150 (78.95)	263
Marginal column totals	186 (100)	190 (100)	376 (grand total)

c	D-dimer > 5000	D-dimer < 5000	Marginal row totals
*Chi-square test of combination of AR poly Q* ≥ *23 and D-dimer value*			
Asn/Asn and AR polyQ ≥ 23	10	19	29
Asp/Asp and Asp/Asn and AR poliQ < 23	40	248	288
Marginal Column totals	50	267	317 (grand total)

*p* value (severe vs mild) = 2.8 × 10^–5^ (OR 2.27, 95% CI 1.54–3.36)

*p* value (severe vs mild) = 1.19 × 10^–4^ (OR 2.42, 95% CI 1.53–3.82)

*p *value (D-dimer > 5000 vs D-dimer < 5000) = 3.73 × 10^–3^ (OR 3.26, 95% CI 1.41–7.52)

The hyper-inflammatory and hyper-thrombotic state, due to viral injury of the vascular endothelium, leads to the release of P-selectin by activated platelets, driving thrombosis and vascular inflammation probably more efficiently in those individuals with enhanced P-selectin activities due a double copy of Asparagine 603 [10]. These results are in line with the demonstration that SARS-CoV-2 induces thrombosis by binding to ACE2 on platelets and subsequent integrin αIIbβ3 activation and P-selectin expression [11], and that P-selectin soluble isoform is increased in thrombosis [6] and severe COVID-19 [7, 8].

Since *SELP* transcription is inhibited by androgens [12], the strength of the association should increase with age. Interestingly, the OR (2.42) in males aged ≥ 50 years with respect to the whole cohort (OR = 2.27) is increased (Table 1).

In a subset of 52 severely affected hospitalised males, four main laboratory parameters related to a proinflammatory state (lymphocyte count, D-dimer and LDH) and a higher risk for thrombosis (D-dimer, platelet count and LDH) were longitudinally followed (Fig. 1b–e). We observed that the maximum pick (over 10 times of the normal upper value) was exclusive of Asp/Asn and Asn/Asn genotypes and older patients (Fig. 1b–e). The pick timing was earlier in Asn/Asn (median 7.5 days from infection) than Asp/Asn (median 13.5 days from infection), (*p* value = 3 × 10^–2^, Fig. 1f). As the vWF is a downstream effector for clotting, the non-0 blood groups, associating with more stable vWF, also correlate with higher D-dimer and LDH values (Fig. 1g, h), in agreement with previous reports [3].

Given the stronger association of the *SELP* polymorphism in older males, the AR poly-Q status would impact on the *SELP* genotype [4]: the combination of poly-Q ≥ 23 with homozygous *SELP* polymorphism versus D-dimer value reached an OR of 3.26 (Table 1c). This result indicates that the two polymorphisms enhance each other, being two pieces of the same puzzle contributing to thrombosis in COVID-19 males.

Anti-*P-Selectin* monoclonal antibodies have been developed for human use: the phase-3 Inclacumab and the FDA&EMA approved Crizanlizumab, the latter as a prevention of vaso-occlusive crises in patients with sickle cell disease [13]. A general clinical trial to test the efficacy and safety of Crizanlizumab in not selected hospitalized COVID-19 patients is ongoing (https://clinicaltrials.gov/ct2/show/study/NCT04435184). Clinical trials in COVID-19 hospitalised males with *SELP* rs6127 should now be encouraged.

## Supplementary Information

**Additional file 1. Material and Methods plus study group appendix.**

## Data Availability

The data are available for sharing through the COVID-19 dedicated section (http://nigdb.cineca.it), within the Network for Italian Genome (http://www.nig.cineca.it). The data and samples referenced here are housed in the GEN-COVID Patient Registry and the GEN-COVID Biobank and are available for consultation. You may contact the corresponding author, Prof. Alessandra Renieri (e-mail: alessandra.renieri@unisi.it).

## Abbreviations

AR: Androgen receptor
SELP: P-selectin gene
vWF: Von Willebrand factor
