# Supplementary material for: SELP Asp603Asn and severe thrombosis in COVID-19 males
Source: J Hematol Oncol. 2021 Aug 16;14:123. doi: 10.1186/s13045-021-01136-9 (PMC8365289; doi:10.1186/s13045-021-01136-9)
Supplement: Supplementary file 1 — Additional file 1. Material and Methods plus study group appendix. [file 13045_2021_1136_MOESM1_ESM.docx]

**MATERIAL AND METHODS**

A cohort of 1,186 SARS-CoV-2-infected subjects was recruited during the first pandemic wave within the Italian GEN-COVID Study, together with detailed clinical and laboratory data (<https://sites.google.com/dbm.unisi.it/gen-covid>) [1].

Separately for the male and female cohorts, two Ordered Logistic Regression (ORL) models were applied using the age to predict the clinical grading according to the WHO Outcome scale [2]. Then, each patient had clinical classification equal to: 0 (mild), if the actual patient grading was below the one predicted by the OLR; or 1 (severe), if the grading was above the OLR prediction. The patients with a predicted gradient equal to the actual gradient were excluded from the LASSO analysis, by which we wanted to compare the “extreme ends” . [3]. The male subset consists of 513 COVID-19 male patients: 236 severe hospitalised COVID-19 patients (severe) and 277 SARS-CoV-2 PCR-positive oligo-asymptomatic not-hospitalised subjects (mild). WES with at least 97% coverage at 20x was performed using the NovaSeq6000 System (Illumina, San Diego, CA, USA) as previously described [1]. WES data were represented in a binary mode on a gene-by-gene basis [3].

LASSO logistic regression model was applied to a synthetic boolean representation under a recessive model of genetic combinations [3]. Association was confirmed by Chi Square Test. A subset of 52 hospitalised males was selected in order to evaluate longitudinal laboratory values related to thrombosis and inflammation: D-dimer (μg/L), platelets count (10^3/mmc), LDH (UI/L) and lymphocytes count (10^3/mmc)). Longitudinal laboratory values were represented by linear graphs. Differences were assessed by Mann–Whitney test and displayed by box plots. Statistical procedures were carried out using R packages.

**References**

1. Daga S, Fallerini C, Baldassarri M, et al. Employing a systematic approach to biobanking and analyzing genetic and clinical data for advancing COVID-19 research. Eur J Hum Genet. Jan 17.doi: 10.1038/s41431-020-00793-7 (2021).
2. WHO R&D Blueprint novel Coronavirus COVID-19 Therapeutic Trial Synopsis. 2020.
3. Nicola Picchiotti, Elisa Benetti, Chiara Fallerini, et al. Post-Mendelian genetic model in COVID-19 medRxiv 2021.01.27.21250593; doi: <https://doi.org/10.1101/2021.01.27.2125059365563>.

**STUDY GROUP APPENDIX**

**GEN-COVID Multicenter Study (**[**https://sites.google.com/dbm.unisi.it/gen-covid**](https://sites.google.com/dbm.unisi.it/gen-covid)**)**

Floriana Valentino^1,2^, Gabriella Doddato^1,2^, Annarita Giliberti^1,2^, Rossella Tita^7^, Sara Amitrano^7^, Mirella Bruttini^1,2,7^, Ilaria Meloni^1,2^, Anna Maria Pinto^7^, Maria Antonietta Mencarelli^7^, Caterina Lo Rizzo^7^, Francesca Montagnani^2,8^, Miriam Lucia Carriero^1,2^, Massimiliano Fabbiani^8^, Ilaria Rancan^8^, Barbara Rossetti^8^, Mario Tumbarello^2,8^, Elena Bargagli^9^, Laura Bergantini^9^, Miriana D’Alessandro^9^, Paolo Cameli^9^, David Bennett^9^, Federico Anedda^10^, Simona Marcantonio^10^, Sabino Scolletta^10^, Federico Franchi^10^, Maria Antonietta Mazzei^11^, Susanna Guerrini^11^, Edoardo Conticini^12^, Luca Cantarini^12^, Bruno Frediani^12^, Danilo Tacconi^13^, Chiara Spertilli Raffaelli^13^, Marco Feri^14^, Alice Donati^14^, Raffaele Scala^15^, Luca Guidelli^15^, Genni Spargi^16^, Marta Corridi^16^, Cesira Nencioni^17^, Leonardo Croci^17^, Gian Piero Caldarelli^18^, Maurizio Spagnesi^19^, Paolo Piacentini^19^, Maria Bandini^19^, Elena Desanctis^19^, Silvia Cappelli^19^, Anna Canaccini^20^, Agnese Verzuri^20^ Valentina Anemoli^20^, Agostino Ognibene^21^, Alessandro Pancrazi^21^, Maria Lorubbio^21^, Massimo Vaghi^22^, Antonella D’Arminio Monforte^23^, Esther Merlini^23^, Federica Gaia Miraglia^23^, Mario U. Mondelli^24,25^, Raffaele Bruno^24,25^, Marco Vecchia^24,^, Stefania Mantovani^24^, Serena Ludovisi^24,25^, Massimo Girardis^26^, Sophie Venturelli^26^, Stefano Busani^26^, Andrea Cossarizza^27^, Andrea Antinori^28^ Alessandra Vergori^28^, Arianna Emiliozzi^28^, Stefano Rusconi^29,30^, Matteo Siano^30^, Arianna Gabrieli^30^, Agostino Riva^29,30^, Daniela Francisci^31^, Elisabetta Schiaroli^31^, Francesco Paciosi^31^, Andrea Tommasi^31^, Francesco Vladimiro Segala^32^, Pier Giorgio Scotton^33^, Francesca Andretta^33^, Sandro Panese^34^, Renzo Scaggiante^35^, Francesca Gatti^35^, Saverio Giuseppe Parisi^36^, Francesco Castelli^37^, Eugenia Quiros-Roldan^37^, Melania degli Antoni^37^, Isabella Zanella^38^, Matteo Della Monica^39^, Carmelo Piscopo^39^, Mario Capasso^40,41,42^, Roberta Russo^40,41^, Immacolata Andolfo^40,41^, Achille Iolascon^40,41^, Giuseppe Fiorentino^43^, Massimo Carella^44^, Marco Castori^44^, Filippo Aucella^45^, Pamela Raggi^46^, Carmen Marciano^46^, Rita Perna^46^, Matteo Bassetti^47,48^, Antonio Di Biagio^47,^aurizio Sanguinetti^49,50^, Luca Masucci^49,50^, Serafina Valente^51^, Oreste De Vivo^51^, Marco Mandalà^52^, Alessia Giorli^52^, Lorenzo Salerni^52^, Patrizia Zucchi^53^, Pierpaolo Parravicini^53^, Elisabetta Menatti^54^, Stefano Baratti^55^, Tullio Trotta^56^, Ferdinando Giannattasio^56^, Gabriella Coiro^56^, Fabio Lena^57^, Domenico A. Coviello^58^, Cristina Mussini^59^, Giancarlo Bosio^60^, Enrico Martinelli^60^, Sandro Mancarella^61^, Luisa Tavecchia^61^, Mary Ann Belli^61^, Lia Crotti^62,63,64,65,66^, Gianfranco Parati^62,63^, Marco Gori^4,67^, Chiara Gabbi^68^, Maurizio Sanarico^69^, Stefano Ceri^70^, Pietro Pinoli^70^, Francesco Raimondi^71^, Filippo Biscarini^72^, Alessandra Stella^72^, Marco Rizzi^73^, Franco Maggiolo^73^, Diego Ripamonti^73^, Claudia Suardi^74^, Tiziana Bachetti^75^, Maria Teresa La Rovere^76^, Simona Sarzi-Braga^77^, Maurizio Bussotti^78^ Simona Dei^79^, Katia Capitani^2,80^, Sabrina Ravaglia^81^, Rosangela Artuso^82^, Antonio Perrella^83^, Francesco Bianchi^2,83^, Davide Romani^19^, Paola Bergomi^84^, Emanuele Catena^84^, Riccardo Colombo^84^, Sauro Luchi^85^, Giovanna Morelli^85^, Paola Petrocelli^85^, Valentina Perticaroli^1,2,7^, Mirjam Lista^1,2^, Silvia Baroni^86^, Alessandra Guarnaccia^49^, Giuseppe Merla^40,87^, Gabriella Maria Squeo^87^

8. Dept of Specialized and Internal Medicine, Tropical and Infectious Diseases Unit, Azienda Ospedaliera Universitaria Senese, Siena, Italy

9. Unit of Respiratory Diseases and Lung Transplantation, Department of Internal and Specialist Medicine, University of Siena

10 Dept of Emergency and Urgency, Medicine, Surgery and Neurosciences, Unit of Intensive Care Medicine, Siena University Hospital, Italy

11. Department of Medical, Surgical and Neurosciences and Radiological Sciences, Unit of Diagnostic Imaging, University of Siena

12 Rheumatology Unit, Department of Medicine, Surgery and Neurosciences, University of Siena, Policlinico Le Scotte, Italy

13. Department of Specialized and Internal Medicine, Infectious Diseases Unit, San Donato Hospital Arezzo, Italy

14. Dept of Emergency, Anesthesia Unit, San Donato Hospital, Arezzo, Italy

15. Cardiothoraco-neurovascular Department, Pulmonology and Respiratory Intensive Care Unit, San Donato Hospital, Usl Toscana Sudest, Arezzo, Italy

16. Department of Emergency, Anesthesia Unit, Misericordia Hospital, Grosseto, Italy

17. Department of Specialized and Internal Medicine, Infectious Diseases Unit, Misericordia Hospital, Grosseto, Italy

18. Laboratory Medicine Department, Misericordia Hospital, Grosseto, Italy

19. Department of Preventive Medicine, Azienda USL Toscana Sud Est, Italy

20. Territorial Scientific Technician Department, Azienda USL Toscana Sud Est, Italy

21. Laboratory Medicine Department, Clinical and Molecular Pathology Sector, Azienda USL Toscana Sudest, Ospedale San Donato, Arezzo, Italy.

22. Chirurgia Vascolare, Ospedale Maggiore di Crema, Italy

23. Department of Health Sciences, Clinic of Infectious Diseases, ASST Santi Paolo e Carlo, University of Milan, Italy

24. Division of Infectious Diseases and Immunology, Fondazione IRCCS Policlinico San Matteo, Pavia, Italy

25. Department of Internal Medicine and Therapeutics, University of Pavia, Italy

26. Department of Anesthesia and Intensive Care, University of Modena and Reggio Emilia, Modena, Italy

27. Department of Medical and Surgical Sciences for Children and Adults, University of Modena and Reggio Emilia, Modena, Italy

28. HIV/AIDS Department, National Institute for Infectious Diseases, IRCCS, Lazzaro Spallanzani, Rome, Italy

29. III Infectious Diseases Unit, ASST-FBF-Sacco, Milan, Italy

30. Department of Biomedical and Clinical Sciences Luigi Sacco, University of Milan, Milan, Italy

31. Infectious Diseases Clinic, Department of Medicine, Azienda Ospedaliera di Perugia and University of Perugia, Santa Maria Hospital, Perugia, Italy

32. Clinic of Infectious Diseases, Catholic University of the Sacred Heart, Rome, Italy.

33. Department of Infectious Diseases, Treviso Hospital, Local Health Unit 2 Marca Trevigiana, Treviso, Italy

34. Clinical Infectious Diseases, Mestre Hospital, Venezia, Italy.

35. Infectious Diseases Clinic, ULSS1, Belluno, Italy

36. Department of Molecular Medicine, University of Padova, Italy

37. Department of Infectious and Tropical Diseases, University of Brescia and ASST Spedali Civili Hospital, Brescia, Italy

38. Department of Molecular and Translational Medicine, University of Brescia, Italy; Clinical Chemistry Laboratory, Cytogenetics and Molecular Genetics Section, Diagnostic Department, ASST Spedali Civili di Brescia, Italy

39. Medical Genetics and Laboratory of Medical Genetics Unit, A.O.R.N. "Antonio Cardarelli", Naples, Italy

40. Department of Molecular Medicine and Medical Biotechnology, University of Naples Federico II, Naples, Italy

41. CEINGE Biotecnologie Avanzate, Naples, Italy

42. IRCCS SDN, Naples, Italy

43. Unit of Respiratory Physiopathology, AORN dei Colli, Monaldi Hospital, Naples, Italy

44. Division of Medical Genetics, Fondazione IRCCS Casa Sollievo della Sofferenza Hospital, San Giovanni Rotondo, Italy

45. Department of Medical Sciences, Fondazione IRCCS Casa Sollievo della Sofferenza Hospital, San Giovanni Rotondo, Italy

46. Clinical Trial Office, Fondazione IRCCS Casa Sollievo della Sofferenza Hospital, San Giovanni Rotondo, Italy

47. Department of Health Sciences, University of Genova, Genova, Italy

48. Infectious Diseases Clinic, Policlinico San Martino Hospital, IRCCS for Cancer Research Genova, Italy

49. Microbiology, Fondazione Policlinico Universitario Agostino Gemelli IRCCS, Catholic University of Medicine, Rome, Italy

50. Department of Laboratory Sciences and Infectious Diseases, Fondazione Policlinico Universitario A. Gemelli IRCCS, Rome, Italy

51. Department of Cardiovascular Diseases, University of Siena, Siena, Italy

52. Otolaryngology Unit, University of Siena, Italy

53. Department of Internal Medicine, ASST Valtellina e Alto Lario, Sondrio, Italy

54. Study Coordinator Oncologia Medica e Ufficio Flussi, Sondrio, Italy

55. Department of Infectious and Tropical Diseases, University of Padova, Padova, Italy

56. First Aid Department, Luigi Curto Hospital, Polla, Salerno, Italy

57. Local Health Unit-Pharmaceutical Department of Grosseto, Toscana Sud Est Local Health Unit, Grosseto, Italy

58. U.O.C. Laboratorio di Genetica Umana, IRCCS Istituto G. Gaslini, Genova, Italy.

59. Infectious Diseases Clinics, University of Modena and Reggio Emilia, Modena, Italy.

60. Department of Respiratory Diseases, Azienda Ospedaliera di Cremona, Cremona, Italy

61. U.O.C. Medicina, ASST Nord Milano, Ospedale Bassini, Cinisello Balsamo (MI), Italy

62. Istituto Auxologico Italiano, IRCCS, Department of Cardiovascular, Neural and Metabolic Sciences, San Luca Hospital, Milan, Italy.

63. Department of Medicine and Surgery, University of Milano-Bicocca, Milan, Italy

64. Istituto Auxologico Italiano, IRCCS, Center for Cardiac Arrhythmias of Genetic Origin, Milan, Italy.

65. Istituto Auxologico Italiano, IRCCS, Laboratory of Cardiovascular Genetics, Milan, Italy.

66. Member of the European Reference Network for Rare, Low Prevalence and Complex Diseases of the Heart-ERN GUARD-Heart

67. University Cote d'Azur, Inria, CNRS, I3S, Maasai

68. Independent Medical Scientist, Milan, Italy

69. Independent Data Scientist, Milan, Italy

70. Department of Electronics, Information and Bioengineering (DEIB), Politecnico di Milano, Milano, Italy

71. Scuola Normale Superiore, Pisa, Italy

72. CNR-Consiglio Nazionale delle Ricerche, Istituto di Biologia e Biotecnologia Agraria (IBBA), Milano, Italy.

73. Unit of Infectious Diseases, ASST Papa Giovanni XXIII Hospital, Bergamo, Italy

74. Fondazione per la ricerca Ospedale di Bergamo, Bergamo, Italy

75. Direzione Scientifica, Istituti Clinici Scientifici Maugeri IRCCS, Pavia, Italy.

76. Istituti Clinici Scientifici Maugeri IRCCS, Department of Cardiology, Institute of Montescano, Pavia, Italy.

77. Istituti Clinici Scientifici Maugeri, IRCCS, Department of Cardiac Rehabilitation, Institute of Tradate (VA), Italy.

78. Istituti Clinici Scientifici Maugeri IRCCS, Department of Cardiology, Institute of Milan, Milan, Italy..

79. Health Management, Azienda USL Toscana Sudest, Tuscany, Italy.

80. Core Research Laboratory, ISPRO, Florence, Italy

81. IRCCS C. Mondino Foundation, Pavia, Italy

82. Medical Genetics Unit, Meyer Children's University Hospital, Florence, Italy

83. Department of Medicine, Pneumology Unit, Misericordia Hospital, Grosseto, Italy.

84. Department of Anesthesia and Intensive Care Unit, ASST Fatebenefratelli Sacco, Luigi Sacco Hospital, Polo Universitario, University of Milan, Milan

85. Infectious Disease Unit, Hospital of Lucca , Italy.

86. Department of Diagnostic and Laboratory Medicine, Institute of Biochemistry and Clinical Biochemistry, Fondazione Policlinico Universitario A. Gemelli IRCCS, Catholic University of the Sacred Heart, Rome, Italy.

87. Laboratory of Regulatory and Functional Genomics, Fondazione IRCCS Casa Sollievo della Sofferenza, San Giovanni Rotondo (Foggia), Italy
